# Mesenchymal and Neural Stem Cell-Derived Exosomes in Treating Alzheimer’s Disease

**DOI:** 10.3390/bioengineering10020253

**Published:** 2023-02-15

**Authors:** Hongmin Wang, Christa C. Huber, Xiao-Ping Li

**Affiliations:** Division of Basic Biomedical Sciences and Center for Brain and Behavior Research, Sanford School of Medicine, University of South Dakota, Vermillion, SD 57069, USA

**Keywords:** Alzheimer’s disease, therapy, therapeutic agent, stem cell, mesenchymal stem cell, neural stem cell, exosome, extracellular vesicle, engineering

## Abstract

As the most common form of dementia and a progressive neurodegenerative disorder, Alzheimer’s disease (AD) affects over 10% world population with age 65 and older. The disease is neuropathologically associated with progressive loss of neurons and synapses in specific brain regions, deposition of amyloid plaques and neurofibrillary tangles, neuroinflammation, blood–brain barrier (BBB) breakdown, mitochondrial dysfunction, and oxidative stress. Despite the intensive effort, there is still no cure for the disorder. Stem cell-derived exosomes hold great promise in treating various diseases, including AD, as they contain a variety of anti-apoptotic, anti-inflammatory, and antioxidant components. Moreover, stem cell-derived exosomes also promote neurogenesis and angiogenesis and can repair damaged BBB. In this review, we will first outline the major neuropathological features associated with AD; subsequently, a discussion of stem cells, stem cell-secreted exosomes, and the major exosome isolation methods will follow. We will then summarize the recent data involving the use of mesenchymal stem cell- or neural stem cell-derived exosomes in treating AD. Finally, we will briefly discuss the challenges, perspectives, and clinical trials using stem cell-derived exosomes for AD therapy.

## 1. Introduction

Alzheimer’s disease (AD) is a progressive neurodegenerative disease strongly associated with aging. The disease gradually impairs learning and memory, problem-solving, language, and other thinking abilities, eventually leading to the loss of the capability to carry out the simplest tasks [1]. AD is the most common form of dementia, accounting for 60–70% of all cases. Other major forms of dementia include vascular dementia, frontotemporal dementia, Lewy body dementia, and mixed dementia [2]. With an increase in the aging population, AD has rapidly become a major public health problem worldwide; analysis of the data obtained from the 1990–2019 Global Burden of Disease database suggests that the incidence and prevalence of AD had increased by 147.95% [3]. According to the Alzheimer’s Association (www.alz.org, accessed on 4 January 2023), over 6 million people currently suffer from AD in the United States alone, spending the nation 321 billion U.S. dollar in 2022. Given the projected trends in population aging, the number of people suffering from AD and the costs associated with this devastating disease are expected to rise.

As AD progresses, brain neurons, including cholinergic neurons, die; accordingly, cholinergic transmission, which plays an important role in learning and memory, would be impaired due to the reduced production of the neurotransmitter acetylcholine [4]. To deal with this problem, cholinesterase inhibitors, such as the United States Food and Drug Administration (FDA)-approved donepezil, rivastigmine, and galantamine, have been clinically used in mild-to-moderate AD patients to block the breakdown of acetylcholine and to increase its availability at synapses. In addition to cholinergic receptors, N-methyl-D-aspartate (NMDA) receptors are also dysregulated. Specifically, excessive activation of NMDA receptors occurs in AD, which also contributes to neuronal death in the disease [5]. Due to this reason, the NMDA receptor antagonist, memantine, an FDA-authorized drug, provides one option to treat moderate-to-severe AD patients [6]. Despite being clinically beneficial in improving and stabilizing symptoms, these medications can only attenuate AD-caused cognitive symptoms for a limited time without stopping or reversing neuronal death [7]. Recently, the FDA-approved aducanumab, a monoclonal antibody, as a therapy for AD. However, the preliminary evidence suggested that this anti-amyloid immunotherapy showed minimal clinical benefits, which is not in proper alignment with its current cost [8]. As a result, developing effective therapies to treat the disease has become urgent.

Increasing data indicate that, as both a therapeutic agent and medicinal delivery vehicle, stem cell-derived exosomes are a promising therapeutic option for AD. In this review, we discuss the neuropathological mechanisms underlying AD and summarize the recent preclinical and clinical data using stem cell-derived exosomes in treating AD.

**Data Sources:** The AD therapy-related citations included in this review were mainly obtained from Pubmed (a total of 88 articles: 46 articles when using key words “Mesenchymal stem cell + exosome + Alzheimer’s disease” for the search, and 22 articles when using “neural stem cell + exosome and Alzheimer’s disease” searched in January 2023, covering the past five years and including research and review articles, published in English.

## 2. Pathology of AD

**Figure 1:**
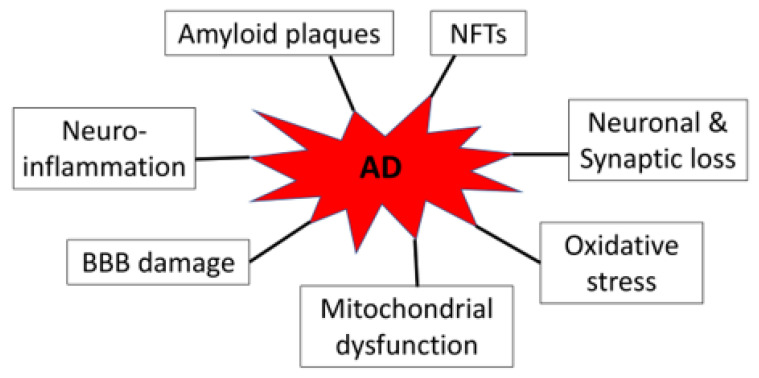
AD-associated neuropathology.

AD is neuropathologically characterized by progressive loss of neurons and synapses, deposition of extracellular amyloid plaques and intracellular neurofibrillary tangles (NFTs), neuroinflammation, blood–brain barrier (BBB) disruption, mitochondrial dysfunction, and oxidative stress (Figure 1). Amyloid plaques and NFTs were initially described by Alois Alzheimer in his seminal 1907 article, yet they are still essential for the postmortem diagnosis of the disease [9]. The amyloid plaques are primarily composed of 36–43 amino acid amyloid-β (Aβ) peptides aggregating in fibrils through enzymatic processing of the amyloid precursor proteins by β- and γ-secretases, while the NFT is mainly made up of hyperphosphorylated tau proteins [10,11]. AD patients can show these pathological alterations years before symptoms of dementia become apparent [12]. Moreover, accumulating data suggest that Aβ and tau proteins are likely driving or causative factors in AD. Even though some individuals who are positive in amyloid and tau protein aggregates in the positron emission tomography studies do not show cognitive impairment, these people are at high risk for future cognitive decline [13].

As the key organelles of energy production in eukaryotic cells, mitochondria play a crucial role in the brain, as the brain requires considerable energy to maintain its various physiological activities. In AD, Aβ aggregates and tau protein fibrils interact with mitochondria and disrupt mitochondrial activities and functions, leading to increased production of reactive oxygen species and oxidative stress [14]. As a result, widespread mitochondrial dysfunction can be detected even in the early stage of AD patient brains [15]. 

Although how neurodegeneration occurs in AD remain unclear, evidence supports that neuroinflammation plays an important role in mediating neuronal and synaptic loss and likely involves the formation of amyloid and tau protein oligomers/fibrils [16]. Indeed, genome-wide association studies have identified multiple immune and microglia-related genes [17,18]. Conversely, blockage of neuroinflammation is considered a preventative strategy and, thus, slows down the progression of AD [16]. Noticeably, in the condition of AD, neuroinflammation is mediated, to a large extent, by microglia, the resident macrophages of the brain and spinal cord [19,20]. Moreover, both Aβ and hyperphosphorylated tau proteins activate microglia in AD and cause the cell to exhibit pro-inflammatory M1 rather than anti-inflammatory M2 phenotypes. M1 phenotypes of microglia, typically induced by interferon-γ and lipopolysaccharide [21], impart the development of neuroinflammation through upregulating pro-inflammatory cytokines and chemokines, including tumor necrosis factor-α, interleukin (IL)-6, IL-1β, IL-12, and CC chemokine ligand 2. Moreover, M1 microglia also produce a lot of reactive oxygen species through the expression of the nicotinamide adenine dinucleotide phosphate oxidase, which also contributes to neuronal injury [22]. In contrast, M2 phenotypes of microglia promote phagocytosis of cell debris and possess anti-inflammatory effects via enhancing the expression of anti-inflammatory molecules, such as IL-10, transforming growth factor-β, insulin-like growth factor-1, fibroblast growth factor, colony stimulating factor-1, nerve-derived growth factor, brain-derived neurotrophic factor, neurotrophins, and glial cell-derived neurotrophic factor, thereby enhancing neuronal survival [21,23]. 

Another AD-related pathological alteration is disruption of the BBB, leading to barrier leakage associated with cognitive decline [24,25]. BBB leakage increases as AD progresses and may contribute to brain aging [26]. Although cerebral amyloid angiopathy characterized by Aβ peptide deposition within the walls of medium to small-caliber blood vessels may contribute to impaired BBB, early inflammation, especially microglia/macrophage as well as B lymphocyte activation, likely mediates BBB hyperpermeability [27]. The role of tau proteins in BBB damage is not well defined and requires further research. Current data are unclear on whether disrupted BBB causes, amplifies, precedes, or coincides with AD [28,29]. It should also be noted that the above-mentioned pathological factors interplay with each other to facilitate AD pathogenesis likely in a combined manner [30].

## 3. Stem Cells and Their Derived Exosomes

### 3.1. Stem Cells

Stem cells are unspecialized cells of the human body that can self-renew and differentiate into any cell type to replace dead or diseased cells. There are two types of stem cells: embryonic stem cells (ESCs) and adult stem cells [31]. ESCs are obtained from abandoned early embryos. Adult stem cells are either directly isolated from fully developed adult tissues, such as connective tissues or stroma, or induced from adult somatic cells. Due to ethical issues of ESCs, adult stem cells, such as a variety of mesenchymal stem cells (MSCs) and induced pluripotent stem cells (iPSCs), have been generated and used in various studies [32,33,34]. Although these MSCs and iPSCs cells are capable of differentiating into specific tissue cell types, the use of these stem cells in different preclinical and clinical neurological studies has yielded mixed results [35]. These inconsistencies could be attributed to the mechanism of action of transplanted stem cells not being well-understood. Moreover, the transplanted MSCs exhibit a low survival rate in the host, and only a small number of cells are seen in the target organ. Importantly, MSCs are dividing cells; therefore, they pose a risk of tumorigenesis. Based on the transplantation studies using different types of stem cells including neural stem cells (NSCs) [36], bone marrow-derived MSCs, and ESCs in treating AD, tumorigenesis was quite a common problem [37]. Moreover, the transplanted stem cells also frequently exhibited poor survival and homing capabilities in these studies. Importantly, the beneficial effect exerted by the transplanted stem cells is largely contributed by the extracellular vesicles, such as exosomes, that contain a variety of growth factors and cytokines to promote brain repair, improve neuronal survival and neurogenesis, and stimulate immunomodulation [38]. Therefore, stem cell-derived exosomes function as effectively as cultured stem cells and overcome many limitations of stem cells. Due to these reasons, stem cell-derived exosomes are increasingly used over stem cells to avoid complications after administration. Notably, after administration, exosomes do not pose a threat to abnormal differentiation and immune rejection, providing a promising therapy for AD [39,40].

### 3.2. Stem Cell-Derived Exosomes

First discovered in the 1980s, exosomes have become a hot topic of research in various disciplines of biomedical sciences, including neurodegeneration and neuroprotection studies. Exosomes are nano-sized single membrane-bound extracellular vesicles generated from the endosomal compartments of various eukaryotic cells [41]. Most eukaryotic cells, including neurons and glia, can secrete exosomes that play important roles in intercellular communication. Exosomes contain mainly proteins, signaling cytokines, lipids, and RNAs that mirror the composition of their donor cells [42]. Accordingly, exosomes derived from stem cells contain different content and play different roles compared to those derived from other types of cells, such as mature neurons or glia.

Biogenesis of exosomes involves late endosomes, where invagination of late endosomal membranes leads to the formation of intraluminal vesicles (ILVs) within the multivesicular body, one type of late endosome. During the biogenesis of exosomes, certain cytosolic and membrane proteins, RNAs, DNAs, lipids, and other components are incorporated into the ILVs. After fusion with the plasma membrane, these ILVs are released from the MVBs into the extracellular space. The ILVs released are termed “exosomes” [42]. The released exosomes can fuse with the plasma membranes of recipient cells through phagocytosis, micropinocytosis, or receptor- or raft-mediated endocytosis. Once exosomes fuse with the recipient cell, the components of exosomes are transferred into the recipient cells. This can occur in nearby cells; alternatively, exosomes can reach distant organs by circulation where they are taken up by the distant recipient organ cells. Exosomes are able to enter into circulation because they are small enough to enter blood circulation by going through the gaps between endothelial cells [43,44]. Alternatively, MVBs can fuse with lysosomes following biogenesis to degrade the contents of the MVBs, including ILVs.

Mounting evidence has suggested that exosomes derived from AD neurons and glia are a major source for the identification of disease biomarkers and pathogenic agents [45]. To discover novel biomarkers for AD, Cai et al. performed a mass spectrometric analysis of circulating plasma exosomes isolated from AD patients or age-matched healthy individuals. This study identified several proteins potentially serving as novel biomarkers to differentiate AD patients from healthy controls [46]. Similar studies were also conducted by other groups [47,48]. On the other hand, as AD patient brain-derived exosomes contain a high level of Aβ, tau protein, and other toxic substances, they are responsible for neuron-to-neuron propagation of these species of proteins [49,50]. Thus, AD brain-derived exosomes not only serve as a major source for the identification of novel biomarkers but also are potential therapeutic targets for the disease.

**Figure 2:**
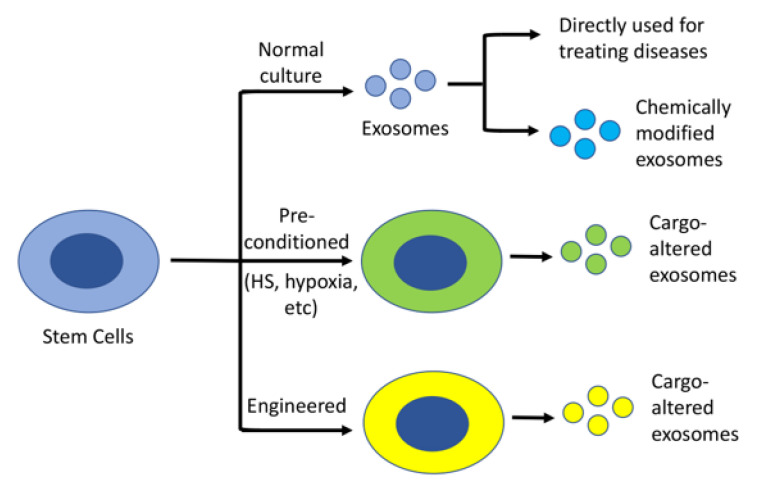
**Stem cell-derived exosomes as a therapeutic agent.** Stem cell-derived exosomes, without or with modification, can be used to treat various diseases. To increase the yield and/or therapeutic efficacy, the cells can be either pre-treated with a specific condition, such as HS or hypoxia, or engineered to overexpress a specific protein.

In contrast to other cell type-derived exosomes, stem cell-derived exosomes possess anti-oxidant, anti-apoptotic, and immunomodulatory properties [51], which offer an equally beneficial effect in comparison to stem cells alone. Many studies have suggested that stem cell-derived exosome-based therapies overcome some of the tricky issues of cell replacement therapy, including apoptosis, necrosis, abnormal differentiation, and tumorigenesis resulting from stress reactions and immune rejection following cell transplantation [52]. In contrast, stem cell-derived exosomes can be isolated from an individual’s own iPSCs or genetically corrected iPSCs, which can then be used to treat various diseases [25]. This is beneficial, as using one’s own cells will not cause an immunogenic issue. Alternatively, the isolated stem cell exosomes can be modified by loading them with additional specific neuroprotective molecules before being used to treat various diseases [53] (Figure 2). One downside to using exosomes is their low yield following isolation. To increase the yield and enhance the therapeutic efficacy of exosomes, stem cell cultures used for collecting exosomes can be pre-conditioned with either heat shock (HS) [54], hypoxia [55,56], or be engineered to [57,58,59] overexpress specific genes before harvesting the medium to collect exosomes (Figure 2). Below, we summarize exosomal isolation techniques and the recent studies using MSC or neural stem cell (NSC)-derived exosomes for treating AD in vitro and in vivo. We also outline the related clinical trials for testing stem cell-derived exosomes in treating the disease. 

### 3.3. Isolation of Exosomes

Various techniques have been established in the past few decades to isolate exosomes from stem cell culture medium or body fluids. This includes ultracentrifugation, size-based filtration, polymer precipitation, and immunoaffinity. A comparison of the advantages and disadvantages of these methods is summarized in Table 1.

#### 3.3.1. Ultracentrifugation

Ultracentrifugation is a traditional method commonly used in isolating stem cell-derived exosomes. This method allows researchers to process a relatively large sample volume. It initially involves using low centrifugal force to remove large debris, followed by high centrifugal force to pellet crude exosomal fractions. The isolated crude exosomes are either directly used in experiments or further subjected to purification by density gradient ultracentrifugation to purify exosomes [25,54].

#### 3.3.2. Size-Based Filtration

Biofluid samples can pass through specific pore-sized filters or size-exclusion chromatography to remove any other extracellular vesicles larger than 150 nm or smaller than 50 nm [60]. This method cannot be used to enrich exosomes. If enrichment of the exosome is needed, ultracentrifugation can be used following these filtration steps.

#### 3.3.3. Polymer Precipitation

Polymer precipitation approaches have been applied to purify exosomes by mixing a polymer, such as polyethylene glycol (PEG), with biofluid samples to capture the vesicles with an exosomal size range (30–150 nm) and reduce exosomal solubility, allowing exosomes to precipitate [61]. This approach relies on the polymer net size and is feasible with routine laboratory equipment.

#### 3.3.4. Immunoaffinity

This method is based on the specific proteins (antigens) located on exosomal membranes. A specific subclass of exosomes can be isolated with high purity using specific antibodies conjugated to a carrier, such as agarose or magnetic beads [62]. Because this method has no volume limitation and can be readily carried out with standard laboratory apparatuses, it is widely used in various applications, including basic research and clinical studies, such as disease diagnosis and prognosis. However, the reagents related to this method are usually expensive.

In addition to the methods mentioned above, other techniques, such as microfluidic-based methods [63], have been developed and utilized for various applications. Microfluidic-based techniques combine sample processing, detection, and analysis into a chip. This innovative method can also be combined with other approaches, making them a low-time-consuming and highly efficient technique.

## 4. Treating AD with Stem Cell-Derived Exosomes

### 4.1. MSC Exosomes in Treating AD

Nearly all tissues harbor MSCs that possess extensive regenerative capabilities and are located in perivascular niches; MSCs can be conveniently obtained from different adult tissues, including bone marrow, adipose tissue, peripheral blood, and neonatal tissues, such as the placenta and umbilical cord [64]. Due to this reason, MSC-derived exosomes have captured great attention in AD therapy studies. Furthermore, current data strongly support that MSC-derived exosomes attenuate AD-related neuropathology improve cognitive performances, and slow down AD pathogenesis in various studies [65,66,67,68,69].

In a cell culture model of AD, Chen et al. found that exosomes directly isolated from MSCs reduced Aβ expression and restored the expression of neuronal memory and synaptic plasticity-related genes [70]. This group also examined the role of MSC-derived exosomes in AD mice and discovered that MSC exosome-treated AD mice showed a significant improvement in brain glucose metabolism and cognitive function compared to the control treatment animals. MSC exosome treatment also reduced Aβ neuropathology and neuronal death. These results were also confirmed by other groups’ studies [69,71]. In addition to alleviation of Aβ-induced cognitive impairment, other groups found that MSC-derived exosomes promoted neurogenesis in the subventricular zone [72] and attenuated neuroinflammation in drug (streptozotocin)-induced AD mice [73], double transgenic (APP/PS1) AD [69,74,75], and triple (APP Swedish, MAPT P301L, and PSEN1 M146V) transgenic AD [68] mouse models. Importantly, MSC-derived exosomes improved AD-like behaviors, including impaired learning and memory behaviors [73,74,75]. In an AD cell culture model, Xiong et al. demonstrated that MSC-derived exosomes reduced the Aβ_42_-induced neurotoxicity [76]. This group further revealed that the neuroprotective effect of the exosomes was likely mediated by the growth differentiation factor-15 (GDF-15) contained in exosomes through the AKT/GSK-3β/β-catenin pathway, as inhibition of this pathway abolished GDF-15-induced neuroprotection [76]. Another study has shown that MSC-derived exosomes contain sphingosine kinase (SphK) and sphingosine-1-phosphate (S1P), and activation of the S1P/SphK pathway might be responsible for their reduction of Aβ deposition and improvement in cognitive function in the double transgenic AD mice [77].

It is noted that MSC-derived extracellular vesicles from three-dimensional (3D) cultures have also been tested in treating AD mice [65,78]. Intranasal administration of these MSC-derived extracellular vesicles to the 5XFAD (5 familial Alzheimer’s disease mutations) mice for four months significantly improved cognitive behaviors and attenuated AD-like neuropathology. Further, these exosomes also reduce Aβ plaque load and exhibit less colocalization between GFAP and Aβ plaques [65].

To improve the therapeutic efficacy of exosomes, pre-treatment of MSCs with hypoxia was demonstrated to confer exosomes a greater beneficial effect on improving cognitive functions and decreasing neuronal damage [79]. Further, exosomes isolated from the hypoxia-pre-treated MSCs effectively shifted hippocampal microglia from the M1 to M2 phenotype in AD mice compared to the control treatments. Similarly, additional studies supported a better therapeutic effect of exosomes derived from the hypoxia-pre-conditioned MSCs than the non-pre-conditioned exosomes in rescuing synaptic dysfunction, suppressing inflammatory responses, and ameliorating cognitive decline in AD mice [67]. Moreover, the therapeutic role of stem cell-derived exosomes is not limited to MSC exosomes; other types of stem cell-derived exosomes, such as NSC-derived exosomes, also show therapeutic effects in AD.

### 4.2. NSC Exosomes in Treating AD

NSC-derived exosomes have also been shown to have a beneficial effect on improving AD-related neuropathology. Treatment of the APP/PS1 double transgenic AD mice with NSC-derived exosomes for five weeks significantly improved their cognitive performance when compared to the control treatment [80]. NSC-derived exosomes also modulated AD-related neuropathology: these exosomes alleviated mitochondrial dysfunction, enhanced Sirtuin activation and synaptic activity, and decreased inflammatory response in mice [80]. Importantly, NSC-derived exosomes also inhibited neuronal apoptosis, possibly by enhancing autophagy via the miRNAs harbored in the exosomes [81]. 

NSC-derived exosomes also improve BBB integrity. Our group examined the role of NSC-derived exosomes in treating AD-caused BBB impairment in a cell culture model. We discovered that NSC-derived exosomes effectively repair the Aβ-induced BBB damage [25]. Our study provided evidence supporting NSC-derived exosomes as a therapeutic agent for AD by enhancing BBB integrity. Finally, our study validated a cell-free assay system that can screen drugs used to treat AD-caused BBB disruption.

Although stem cell-derived exosomes have immense therapeutic potential as an AD therapy, the yield of exosomes from cultured stem cell media is usually very limited, which poses a significant restriction to their applications as a therapeutic agent [82,83,84]. Thus, a variety of attempts have been made to improve the yield of exosomes, including exposure of cell culture to hypoxia [56], HS [54], other conditions [83], or overexpression of a specific protein [58]. We recently tested the effect of pre-treatment of NSCs with HS on the yield and therapeutic potential of AD and discovered that NSCs exposed to 42 °C for three hours caused a dramatic increase in the yield of exosomes [54]. Interestingly, HS also enlarged the diameter of exosomes compared to non-heat shock (NHS) cell-derived exosomes. Specifically, the concentration of exosomes isolated from the HS-pre-treated NSCs was 13 times higher than NHS-derived exosomes, even though the proteins of HS-derived exosomes analyzed by mass spectrometry showed decreased diversity when compared to those NHS-derived exosomes. Nevertheless, the bioinformatic analysis of these proteins suggested that the top two biological functions in HS-derived exosomes were negative regulation of apoptotic process and DNA damage, indicating that these exosomes might have specific therapeutic efficacy against apoptotic cell death. To validate their therapeutic potential, exosomes were tested in AD cell culture models. Neuronal cells were incubated with hydrogen peroxide or Aβ_1–42_ in the presence of HS- or NHS-derived exosomes. Our results demonstrated that cells incubated with HS-derived exosomes showed a greater beneficial effect against hydrogen peroxide and Aβ-induced neurotoxicity compared with the NHS-derived exosomes [54]. These results indicate that HS enhances the production of exosomes in NSCs without reducing their therapeutic efficacy in AD.

### 4.3. Preclinical AD Models Used to Test the Therapeutic Efficacy of Stem Cell-Derived Exosomes

To validate the therapeutic efficacy of stem cell-derived exosomes, both cell culture and animal models of AD have been used in various studies. The cell culture models of AD usually involve treating neuronal cells with the toxic Aβ_1–42_ peptide [54,76]. For AD animal models, the most frequently used models are several transgenic mice, including the double transgenic mice [67,69,74,85], triple transgenic mice [68], and 5XFAD transgenic mice [65] that overexpress specific mutant familial AD genes, such as mutant amyloid precursor protein, presenilin-1 or -2, or tau proteins, although drug-induced AD models [73] are also established. 

Despite significant improvement in exosomal purity and efficacy, efficiently delivering exosomes to a specific brain region remains a challenge. Currently, there are four major administration routes for AD therapies in animals: intravenous injection [86], oral administration [87], stereotactic injection [88], and intranasal spray [65]. The most widely used route of administration for therapeutic exosomes is intravenous injection. However, exosomes in blood circulation are readily cleared by macrophages, resulting in a short half-life (from several minutes to a few hours) [89]. In addition, exosomes administered by this route have off-target effects, where they are more likely to go to the kidneys, liver, and spleen upon administration. Due to these reasons, oral administration remains an important method for delivering exosomes. Oral administration enables less fluctuation of exosome levels in plasma following administration. Moreover, this method is convenient and less time-consuming [87]. The most evident advantage of stereotactic injection is that it can directly deliver exosomes to the target site without causing off-target problems. At the same time, it is invasive and requires a high operative skill to conduct the procedure. On the other hand, intranasal delivery of exosomes has become a promising efficient delivery method because it allows exosomes to bypass the BBB to reach the brain conveniently [90].

### 4.4. Therapeutic Mechanisms of Stem Cell-Derived Exosomes

How stem cell-derived exosomes regulate AD-related neuropathology and confer neuroprotection remains to be defined. Given the various molecules exosomes carry, the neuroprotective mechanisms involved are likely multiple, including anti-inflammatory, immunomodulatory, anti-oxidative, anti-apoptotic, and anti-aging effects [91,92,93]. Stem cell-derived exosomes contain various immunomodulatory factors, including prostaglandin E2, hepatic growth factor, transforming growth factor-β, indolamine 2,3-dioxygenase-1, and IL-10 [94]. These factors confer anti-inflammatory and immunomodulatory effects by suppressing reactive astrocytes and activated microglia. Exposure of stem cells to a stressful condition can dramatically alter exosomal contents, thereby changing their functions. For instance, following HS treatment of NSCs, the top-ranked biological function of the exosomal proteins was changed to “anti-apoptotic” in our studies [54]. On the other hand, stem cell-derived exosomes contain specific proteases, such as neprilysin and matrix metalloproteinases, that degrade intracellular and extracellular Aβ [70]. The anti-aging effect of stem cell-derived exosomes is reflected by the promotion of neurogenesis [95]. These different mechanisms are likely to provide a synergistic role against AD pathology, given the interplay between each mechanism.

In addition to the immunomodulatory and neurotrophic factors, stem cell-derived exosomes contain miRNAs, such as miRNA-146a, miRNA-29b, and miRNA-223, which may also mediate anti-inflammatory effects [96,97,98]. These miRNAs can reduce Aβ-induced toxicity and have anti-inflammatory and anti-apoptotic effects.

## 5. Stem Cell Exosomes in AD Clinical Trails

Although a database search of the ClinicalTrials.gov (accessed on 4 January 2023) website resulted in 38 studies using the keywords “stem cell exosome”, there is only one Phase I/II clinical trial listed as #NCT04388982 on the website when “Alzheimer disease” was added to the “Condition or disease” field during the search. This AD clinical trial aims to test the safety and efficacy of exosomes derived from allogenic adipose MSCs in treating mild to moderate AD-caused dementia. Three different doses of MSC exosomes, including low (5 µg), medium (10 µg), and high (20 µg), were proposed. This trial will include at least participants during the 12-week study. These participants will receive exosomes as a nasal drop. In addition, the liver or kidney functions and cognitive performance will be measured to determine any adverse effects of the treatment. Although the study was estimated to be completed in August 2022, the results from the study have yet to be posted. Given the variety of promising results obtained from other clinical studies involving the use of stem cell-derived exosomes in other disorders, such as those in treating gastrointestinal fistulas [99], COVID-19 infections [100], SARS-CoV-2 infection [101], and chronic kidney disease [102], clinical trials using stem cell-derived exosome to treat AD will likely offer promising results.

## 6. Challenge and Perspective

One challenge the field faces is obtaining a high enough yield of stem cell-derived exosomes with high purity while still maintaining the original cargo composition. This is crucial for their clinical applications. Secondly, different exosomal isolation methods involve the use of different reagents, which are likely contaminants of the isolated exosomal fractions. These contaminants and various protein contaminations derived from the biofluids used for the isolation may influence exosomal targeting and functions, resulting in reduced therapeutic efficacy and making it difficult to determine the real effect of exosomes in specific studies [103,104]. Thirdly, because the therapeutic efficacy of exosomes is highly dependent on the cell type, physiological condition, and age of the stem cells that produce the exosomes, standardizing specific stem cell cultures used for isolating exosomes is also a challenge. Finally, the optimal clinical dosage, administration routes, and side effects of long-term administration of stem cell-derived exosomes remain issues to be resolved.

Future research is needed to overcome these challenges to improve the therapeutic efficacy and successfully translate the related research results from the bench to the bedside. Moreover, standardized protocols for the isolation of exosomes should be developed to increase their purity while maintaining biologically active cargo contents. As the route and frequency of administration may greatly influence dosage, an optimal treatment scheme, at least some type of principle related to this, should be established in the future. Further, although tetraspanins CD9, CD63, and CD81 are frequently used as biomarkers in exosome isolation, they cannot be reliably applied in immunoaffinity isolation of exosomes because the expression of these proteins varies significantly in various types of cells [105]. Therefore, it is needed in future research to identify common biomarkers for exosomes isolated from different types of cells, including stem cells.

## 7. Conclusions

AD is a major health problem in our society and is associated with several major neuropathologically features. Accumulating data suggest that exosomes derived from stem cells, such as MSCs or NSCs, are promising therapeutic agents for the treatment of various diseases and have been increasingly tested across multiple AD models. A variety of culture conditions and engineered approaches can increase the yield of exosomes and enhance their therapeutic efficacy. A standard guideline for manufacturing exosomes should be established to conduct high-quality preclinical and clinical studies.

## Figures and Tables

**Table 1:** Comparison of exosome isolation methods.

Methods	Advantages	Disadvantages
Ultracentrifugation	Straightforward, inexpensive, suitable for large sample volumes	Time-consuming, low yield, medium purity
Size-Based Filtration	No limit for sample volumes, no need of special equipment or reagents, highly reproducible, high yield	Deformation of exosomes
Polymer Precipitation	Simple, suitable for large sample volume	Low purity
Immunoaffinity	Simple, high purity	Time-consuming, low yield, expensive

## Data Availability

Not applicable.
